# Phosphorus and sulfur SAD phasing of the nucleic acid-bound DNA-binding domain of interferon regulatory factor 4

**DOI:** 10.1107/S2053230X21006506

**Published:** 2021-06-29

**Authors:** Alessandro Agnarelli, Kamel El Omari, Ramona Duman, Armin Wagner, Erika J. Mancini

**Affiliations:** a School of Life Sciences, University of Sussex, Falmer, Brighton BN1 9QG, United Kingdom; b Diamond Light Source, Harwell Science and Innovation Campus, Didcot OX11 0DE, United Kingdom; c Research Complex at Harwell, Rutherford Appleton Laboratory, Didcot OX11 0FA, United Kingdom

**Keywords:** experimental phasing, native SAD, phosphorus, DNA, DNA-binding proteins, IRF4, interferon regulatory factor 4

## Abstract

Pivotal to the regulation of key cellular processes such as the transcription, replication and repair of DNA, DNA-binding proteins play vital roles in all aspects of genetic activity. The determination of high-quality structures of DNA-binding proteins, particularly those in complexes with DNA, provides crucial insights into the understanding of these processes. The presence in such complexes of phosphate-rich oligonucleotides offers the choice of a rapid method for the routine solution of DNA-binding proteins through the use of long-wavelength beamlines such as I23 at Diamond Light Source. This article reports the use of native intrinsic phosphorus and sulfur single-wavelength anomalous dispersion methods to solve the complex of the DNA-binding domain (DBD) of interferon regulatory factor 4 (IRF4) bound to its interferon-stimulated response element (ISRE). The structure unexpectedly shows three molecules of the IRF4 DBD bound to one ISRE. The sole reliance on native intrinsic anomalous scattering elements that belong to DNA–protein complexes renders the method of general applicability to a large number of such protein complexes that cannot be solved by molecular replacement or by other phasing methods.

## Abstract 2

Solution of the structure of the DNA-binding domain of interferon regulatory factor 4 bound to its interferon-stimulated response element by native intrinsic phosphorus and sulfur single-wavelength anomalous dispersion methods (native SAD) is described.

## 1. Introduction   

DNA-binding proteins are essential components of all bio­logical systems, where they perform crucial roles. Deregulation or mutation of DNA-binding proteins, such as transcription factors, is closely associated with the pathogenesis of several human diseases, including cancer, making them attractive therapeutic targets (Lee & Young, 2013 ▸; Hudson & Ortlund, 2014 ▸). Structure solution of protein–DNA complexes provides the basis of our understanding of normal and pathogenic DNA metabolism and underpins attempts to develop novel drugs targeting disease-associated DNA-binding proteins (Bushweller, 2019 ▸). The last ten years have witnessed a step-change increase in the number of experimentally determined protein–nucleic acid complexes. More than two thirds of all structures of complexes deposited in the Protein Data Bank (PDB) as of April 2021 (6145 out of 9204) were solved in the last ten years. However, the number of protein–nucleic acid complex structures solved remains only a small part of the deposited structures as their experimental determination often remains challenging. The lack of suitable homologous structures can be an obstacle to solving the crystallographic phase problem. Even when molecular replacement (MR) can be employed, DNA-binding proteins can be flexible and/or disordered (Munshi *et al.*, 2018 ▸; Dyson & Komives, 2012 ▸; Varadi *et al.*, 2015 ▸), whilst nucleic acids can depart from canonical structures (Tateishi-Karimata & Sugimoto, 2020 ▸). Occasionally, multiple conformational folds are displayed, producing highly dynamic structural ensembles (Fuxreiter *et al.*, 2011 ▸). As a result, MR alone can often deliver electron-density maps that are of poor quality and are unsuitable for model building and structure solution.

Experimental phasing sidesteps the lack of homologous structures and helps in obtaining interpretable electron-density maps; however, the artificial addition of anomalous scatters by heavy-atom derivatization or selenomethionine substitution can be a time-consuming and often arduous task. On the other hand, native single-wavelength anomalous dispersion (SAD) phasing using the weak anomalous scattering signal of light atoms that are intrinsically present in proteins and nucleic acids, such as phosphorus, sulfur, chlorine, potassium and calcium, obviates the need for covalent or noncovalent heavy-atom modifications. In comparison with metals, however, the anomalous scattering signal from these light atoms is relatively small, and native SAD phasing is critically dependent on accurate recording (Rose & Wang, 2016 ▸). The challenges associated with native SAD phasing are illustrated by the observation that whilst the first native SAD structure was reported in 1981 (Hendrickson & Teeter, 1981 ▸), it took almost 20 years for more structures to be solved (Dauter *et al.*, 1999 ▸; Liu *et al.*, 2000 ▸) by using solvent-flattening approaches (Wang, 1985 ▸). Over the past 20 years, advances in hardware, software, data-collection methods and strategies have allowed the collection of highly accurate data with an increase in the anomalous signal-to-noise ratio, which in turn has enabled the ‘routine’ use of native SAD phasing for *de novo* structure solution (Rose *et al.*, 2015 ▸). Furthermore, the use of native SAD phasing, for example from S atoms, has been successfully used in combination with MR (MRSAD) to overcome model bias and assist with phasing, model building and refinement (Schuermann & Tanner, 2003 ▸).

**Figure 1:**
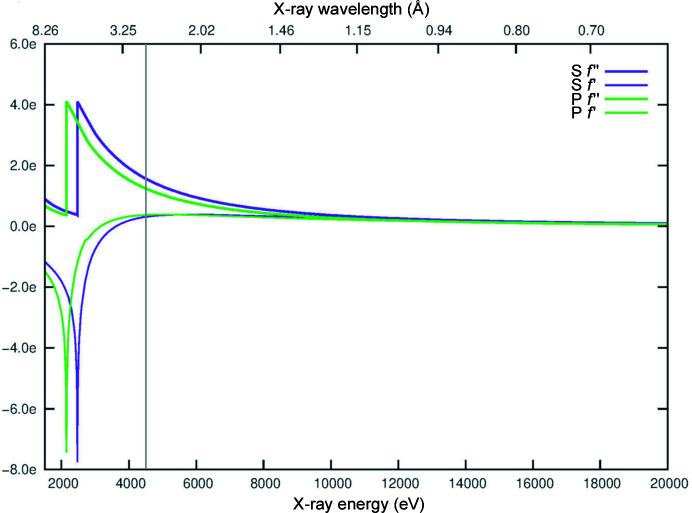
Theoretical values of *f*′ and *f*′′ for the elements sulfur (purple) and phosphorus (green) over energies from 1.5 to 20 keV. The grey bar indicates the wavelength/energy (2.7552 Å/4.5 keV) at which the IRF4 DBD–ISRE DNA data sets were collected. The plot was generated using the http://www.bmsc.washington.edu/scatter website.

The use of the intrinsic anomalous signal of phosphorus to phase oligonucleotide crystal diffraction data using SAD (P-SAD) was first theoretically and practically demonstrated in 2001 (Dauter & Adamiak, 2001 ▸). However, when not in complex with proteins, phasing of nucleic acid structures using P-SAD is very challenging and has in effect been limited to a very small number of cases where crystals diffracted to high resolution (Raiber *et al.*, 2015 ▸; Luo *et al.*, 2014 ▸). There are two possible explanations for the lack of success of P-SAD on nucleic acid structures: the high mobility and consequent high *B* factors of P atoms in the nucleic acid backbone (Harp *et al.*, 2016 ▸) and the reduced number of reflections available for phasing compared with the large number of P atoms (typically small unit cells and often high-symmetry space groups). Lower *B* factors and a higher ratio of reflections to sites in the substructure have been shown to be crucial for SAD phasing in general (Terwilliger *et al.*, 2016 ▸). On the other hand, because interactions with proteins usually stabilize nucleic acid backbones and the number of reflections is greater in larger unit cells, P-SAD can be routinely used for phasing protein–nucleic acid complexes as long as the anomalous signal can be precisely retrieved. The level of difficulty of extracting the intrinsic anomalous signal at in-house or synchrotron beamline wavelengths can be appreciated from a graph of *f*′ and *f*′′ of phosphorus, as seen in Fig. 1 ▸. In practice, the signal-to-noise ratios necessary to adequately and routinely retrieve the anomalous signal of phosphorus are achievable only with very high multiplicity data or at wavelengths that are only obtainable at state-of-the-art long-wavelength beamlines such as I23 at Diamond Light Source. This beamline operates under vacuum with a large semi-cylindrical detector (PILATUS 12M, Dectris) to minimize absorption effects and allow measurements of larger diffraction angles at longer wavelengths (Wagner *et al.*, 2016 ▸).

Here, the structure of the DNA-binding domain (DBD) of interferon regulatory factor 4 (IRF4) bound to its interferon-stimulated response element (ISRE), solved by the use of native intrinsic phosphorus and sulfur single-wavelength anomalous dispersion methods at I23, is presented. The structure shows the presence of three molecules of the IRF4 DBD bound to one molecule of DNA, which is unexpected in the light of previous studies suggesting the homodimerization of IRF4 on ISRE elements (Ochiai *et al.*, 2013 ▸). This study suggests that native intrinsic SAD methods can be used successfully and routinely on long-wavelength beamlines such as I23 to solve protein–nucleic acid structures *de novo*, eliminating the need for molecular replacement.

## 2. Materials and methods   

### 2.1. Protein expression and purification   

The IRF4 DBD (amino acids 20–139) was cloned into a pCDFDuet-1 bacterial expression plasmid containing an N-terminal 6×His tag, transformed into the *Esherichia coli* BL21 strain (Novagen) and grown at 310 K by shaking at 180 rev min^−1^ in Luria–Bertani (LB) broth until the absorbance at 600 nm reached a value of 0.6. Overexpression of the fusion protein was induced by the addition of 0.4 m*M* isopropyl β-d-1-thiogalactopyranoside (IPTG) and growth was continued for 16 h at 291 K. The cells were harvested by centrifugation, resuspended in lysis buffer [25 m*M* HEPES pH 7.4, 150 m*M* NaCl, 5 m*M* imidazole, 0.1 m*M* MgCl_2_, 0.01% Triton X-100, 0.5 m*M* tris(2-carboxyethyl)phosphine (TCEP), protease-inhibitor cocktail] and lysed by sonication on ice. The lysate was clarified by centrifugation at 26 700*g* for 45 min at 277 K. The supernatant was applied onto a HisPur Cobalt Resin column (Thermo Fisher) previously equilibrated with wash buffer (25 m*M* HEPES pH 7.4, 150 m*M* NaCl, 5 m*M* imidazole, 0.5 m*M* TCEP). Following a 10 min incubation at 227 K and the application of five column volumes of wash buffer, the protein was eluted by the addition of elution buffer (25 m*M* HEPES pH 7.4, 150 m*M* NaCl, 150 m*M* imidazole, 0.5 m*M* TCEP). The collected eluate was concentrated and purified by size-exclusion chromatography using a HiLoad 16/600 Superdex 75 prep-grade column (GE Healthcare) in gel-filtration buffer (20 m*M* Tris–HCl pH 8, 500 m*M* NaCl, 0.5 m*M* TCEP) at 277 K. Fractions were analysed on a 14% SDS–PAGE gel by electrophoresis and those containing the IRF4 DBD were pooled and concentrated to 10 mg ml^−1^. Oligonucleotides containing an interferon response element (ISRE), 5′-AATAAAAGAAACCGAAAGTAA-3′ and 5′-TTTACTTTCGGTTTCTTTTAT-3′ (Eurofins Genomic), were annealed and incubated in a 1.2:1 DNA:protein molar ratio for 1 h at 277 K prior to crystallization.

### 2.2. Crystallization   

The IRF4 DBD–ISRE complex was used to screen 384 conditions using the sitting-drop vapour-diffusion method. Initial hits appeared within a week and were optimized with an additive screen (JBScreen Plus HTS). The best crystals grew in 0.1 *M* sodium acetate pH 5.2, 5% PEG 4000, 10 m*M* EDTA at 293 K. Crystals were harvested using sample holders designed specifically for experiments on the in-vacuum I23 beamline and were successfully cryoprotected in 25% glycerol by flash-cooling in liquid nitrogen.

### 2.3. Data collection and processing   

Diffraction data from two crystals of the IRF4 DBD–DNA complex were collected on a PILATUS 12M detector (Dectris) at ∼60 K on the long-wavelength beamline I23 at Diamond Light Source, Didcot, UK (Wagner *et al.*, 2016 ▸). From each crystal, four data sets of 360° (rotation increment 0.1°, exposure 0.1 s) were collected with different κ and φ angles at a wavelength of 2.7552 Å (energy 4.5 keV). The eight data sets were each processed independently with *XDS* and then merged together with *XSCALE* (Kabsch, 2010 ▸) in space group *C*222_1_. Intensities were subsequently scaled to amplitudes in *AIMLESS* (Evans & Murshudov, 2013 ▸).

### 2.4. Structure solution and refinement   

Structure solution was performed using native SAD techniques. The automatic experimental phasing pipeline *Crank*2 (Skubák & Pannu, 2013 ▸) using *PRASA* with 20 000 trials and a resolution cutoff of 3.2 Å found a substructure of 39 atoms with an occupancy of at least 25%. The pipeline provided an interpretable electron-density map and a starting model in which three IRF4 DBD molecules could be identified. The electron-density map quality and the location of the phosphorus sites allowed the manual building of the double-stranded DNA, since the pipelines is not yet able to build nucleic acids, and improvement of the IRF4 DBD molecules in *Coot* (Emsley *et al.*, 2010 ▸). Refinement was carried out with *phenix.refine* (Liebschner *et al.*, 2019 ▸) with a strategy consisting of positional, individual *B* factor, TLS and NCS refinement. The final IRF4 DBD–DNA complex structure was refined to 2.6 Å with an *R*_work_ and *R*_free_ of 21.2% and 24.1%, respectively, and was validated with *MolProbity* (Chen *et al.*, 2010 ▸). The final refined structure is composed of three molecules of IRF4 (residues 21–134, 22–130 and 19–130, respectively) and the 21 base pairs of ISRE DNA. Data-collection and refinement details are presented in Table 1 ▸.

## 3. Results   

The human IRF4 DBD domain was expressed, purified and co-crystallized with 21-mer DNA with an AT 5′ overhang containing an ISRE element. Diffraction data were initially collected at a wavelength of 0.9795 Å on beamline I04 at Diamond Light Source. A complete data set was collected to a resolution limit of 2.75 Å from a crystal belonging to space group *C*222_1_, with unit-cell parameters *a* = 78.2, *b* = 112.5, *c* = 139.4 Å. The identification of the content of the crystal asymmetric unit via analysis of the Matthews coefficient was not unambiguous. The most likely oligomeric state, as suggested by previous studies (Ochiai *et al.*, 2013 ▸), is that of an IRF4 homodimer bound to one ISRE element, suggesting a molecular weight for the complex of about 44.8 kDa. The volume of the crystal asymmetric unit is compatible with the presence of either one (*V*_M_ = 3.4 Å^3^ Da^−1^, solvent content 64%) or two (*V*_M_ = 1.7 Å^3^ Da^−1^, solvent content 28%) copies of such a complex. Initial attempts to solve the structure by molecular replacement using the NMR structure of the IRF4 DBD (PDB entry 2dll; RIKEN Structural Genomics/Proteomics Initiative, unpublished work) to search for either one or two copies of the complex were unsuccessful. Automatic molecular-replacement programs such as *Phaser.MRage* (Bunkóczi *et al.*, 2013 ▸), where the asymmetric unit content can be left for the program to establish even when the number of copies of a single component are unknown, were also unsuccessful. Several reasons including conformational differences between the model and the data or inherent inaccuracies in the NMR model could account for the failure of this approach.

Taking advantage of the dedicated long-wavelength beamline I23 at Diamond Light Source, data were collected at a wavelength of 2.7552 Å with the aim of solving the structure of the complex *de novo* using native intrinsic phosphorus and sulfur SAD methods. The wavelength choice, guided by the experience of previous successful experiments on beamline I23, is a compromise between anomalous signal strength and absorption effects that decrease the data quality. Absorption increases with the cube of the wavelength and although in a high-vacuum environment there is no air absorption, absorption by the crystal, the sample holder and the surrounding mother liquor together can have a severe impact on the recorded intensities at long wavelengths. The impact on intensities is further exacerbated if the X-ray path length varies significantly depending on the crystal orientation. The limitations of native SAD phasing experiments that use wavelengths longer than 3 Å have previously been described (Basu *et al.*, 2019 ▸). At a wavelength of 2.7552 Å, S and P atoms contribute with anomalous differences *f*′′ of 1.6 e and 1.3 e, respectively (Fig. 1 ▸). The final high-multiplicity (∼80) data set to a resolution of 2.6 Å (space group *C*222_1_, unit-cell parameters *a* = 77.9, *b* = 112.4, *c* = 140.7 Å) was obtained by merging eight data sets collected from two crystals: four data sets from each crystal.

**Figure 2:**
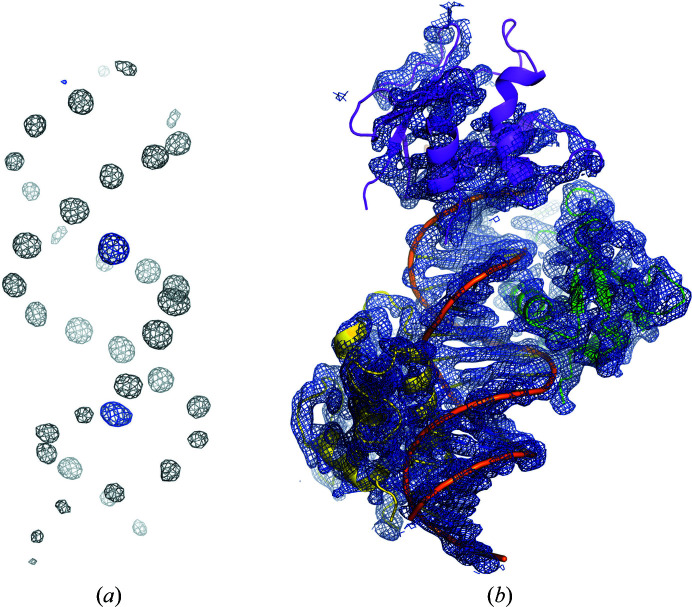
Difference Fourier anomalous map and experimental electron-density map for the IRF4 DBD. (*a*) Difference Fourier anomalous map contoured at 5σ generated by *Crank*2 from the partially built model (no nucleic acids built). The grey electron density corresponds to P atoms from the DNA molecule, and the blue electron density, in the major DNA groove, to the S atoms from the IRF4 DBD. The weaker electron density at the very top of the picture corresponds to the S atom from the third IRF4 DBD molecule. (*b*) Experimental electron-density map generated by *Crank*2. The final model of the IRF4 DBD is fitted in the map to assess the map quality. This figure was prepared with *PyMOL* (version 2.0; Schrödinger).

**Figure 3:**
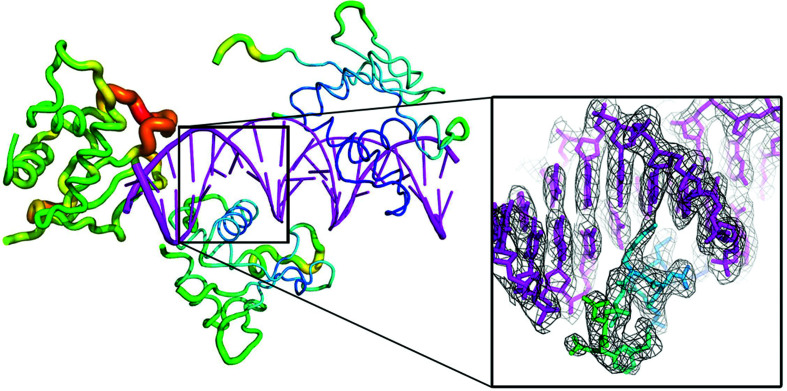
Crystal structure of the IRF4 DBD on ISRE DNA. The IRF4 DBD is in a *B*-factor putty cartoon representation, where the cartoon thickness and colour reflect the relative C^α^ *B* factors within the molecule. The ISRE DNA is coloured magenta. The σ_A_-weighted 2*F*_o_ − *F*_c_ refined map is shown (grey mesh) at a contour level of 1.5σ. The map, focusing on one of the recognition helices, was carved around the atomic model of the IRF4 DBD with a border of 2 Å to improve clarity. This figure was prepared with *PyMOL* (version 2.0; Schrödinger).

*PRASA*, as part of the automatic structure-determination pipeline *Crank*2, was able to locate 39 atoms of the substructure within which the DNA double helix could be readily recognized (Fig. 2 ▸ *a*). The substructure was used to produce an interpretable electron-density map in which, surprisingly, three IRF4 DBD molecules were identified (Fig. 2 ▸ *b*). The quality of the electron-density map and the phosphorus sites were instrumental in the manual building of the DNA oligonucleotide since the *Crank*2 pipeline does not support the automatic building of nucleic acids. Iterative cycles of manual model building with *Coot* and refinement with *phenix.refine* allowed full structure determination. The final model contained three molecules of the IRF4 DBD bound to one ISRE element, which fits well in the crystallo­graphic asymmetric unit (*V*_M_ = 2.5 Å^3^ Da^−1^, solvent content 51%) based on an estimated molecular mass of ∼61 kDa. When calculating phased anomalous difference maps with *ANODE* (Thorn & Sheldrick, 2011 ▸), the three IRF4 DBD sulfur sites gave anomalous peaks that were stronger on average than the DNA phosphorus sites (∼11σ versus ∼9σ); however, one of the sulfur sites had a much lower peak height when compared with the other two sites (∼4σ compared with ∼18σ and 13σ) (Fig. 2 ▸ *a*). The corresponding IRF4 DBD molecule displays poorly defined electron density and higher *B* factors when compared with the other two IRF4 DBD molecules in the asymmetric unit (167 Å^2^ when compared with 87 and 112 Å^2^), as shown in Fig. 3 ▸.

Previous studies using electrophoretic mobility shift assays suggested that IRF4 binds the ISRE element as a homodimer with low affinity (Ochiai *et al.*, 2013 ▸). The finding of three molecules bound to ISRE is unexpected, and a full structural and biophysical analysis of binding affinities is currently under way.

## 4. Discussion   

Although native SAD remains a challenging method for the solution of nucleic acid crystal structures (Harp *et al.*, 2016 ▸), this is not the case for protein–nucleic acid complexes. Native intrinsic phosphorus and sulfur SAD was chosen as a fast and elegant method for the determination of the IRF4 DBD–DNA complex structure. This technique does not rely on selenomethionine substitution or heavy-atom derivatization, but instead measures the anomalous signal from light atoms that are naturally present in proteins and nucleic acids. As the error associated with the measurement decreases with the square of the number of observations, a high-multiplicity data set was obtained by collecting and merging eight diffraction data sets collected from two different crystals. The individual data sets were collected at different κ and φ angles to minimize systematic error due to the experimental setup. As the I23 beam is unfocused, the beam flux is reduced, allowing multiple sweeps of 360° of data to be collected at low dose using the settings described in Section 2. Data collection is brought to an end when signs of radiation damage are detected, either via a decrease in the anomalous signal resolution or a decrease in the number of reflections recorded during data collection, by using *Diffraction Image Screening Tool and Library* (*DISTL*) software plots (Zhang *et al.*, 2006 ▸). The merging of the data sets increases the Bijvoet multiplicity at the same time as limiting the radiation damage. The higher redundancy increases the accuracy of the data and the strength of the anomalous signal to noise of the data set (Liu *et al.*, 2012 ▸).

Of the 45 anomalous scatterers in the asymmetric unit (42 P atoms in the double-stranded DNA and one S atom per IRF4 DBD molecule), 39 could be initially identified by *PRASA*, providing sufficient anomalous signal to phase the whole complex. Of the three S atoms, however, one produced a very weak anomalous signal when compared with the other two. This sulfur belongs to an IRF4 DBD molecule with poorly defined electron density and higher *B* factors. With only two strong anomalous sulfur sites, it could be argued that the structure of this specific complex could have been solved using the phosphorus substructure alone. A further advantage of solving the phosphorus substructure was that the DNA double helix was readily recognisable and the electron-density map for the nucleic acid portion of the structure was strong. The phosphorus sites were used as well defined guides for fitting and building the DNA double-helix model, which is important when, as in this case, the DNA departs from the canonical B form (Fig. 3 ▸).

Native intrinsic SAD phasing is particular helpful when homologous models for molecular replacement are not available, when molecular replacement is not successful and/or when the initial electron-density maps are not suitable for model building. At the time of this study, only an NMR model of the IRF4 DBD domain was available as a molecular-replacement model and it did not lead to a clear phasing solution. The molecular-replacement procedure was confounded by the unexpected oligomerization state of the complex: a heterotetramer with three IRF4 DBD molecules bound to one ISRE element. Furthermore, one of the three IRF4 DBD molecules in the asymmetric units displayed poor electron density and high *B* factors (Fig. 3 ▸), which might also explain the difficulty in solving the structure of the complex by molecular replacement.

Despite the challenges associated with the technique, native SAD phasing is on the brink of becoming the routine method of choice for *de novo* structure determination (Rose *et al.*, 2015 ▸). The availability of dedicated long-wavelength beamlines to increase the anomalous scattering signal of intrinsic light atoms has been instrumental in the increasing popularity of the method. Protein–DNA complexes are especially good candidates for native SAD phasing at long wavelengths since the technique is particularly suited for sulfur and phosphorus substructure detection. To conclude, this work suggests that by using long-wavelengths beamlines, such as I23 at Diamond Light Source, this method could be generally applicable to a large number of nucleic acid–protein complexes.

## Supplementary Material

PDB reference: DNA-binding domain of interferon regulatory factor 4, 7o56

## Figures and Tables

**Table 1:** Data-collection and refinement statistics Values in parentheses are for the highest resolution shell.

Wavelength (Å)	2.755
No. of crystals	2
Resolution range (Å)	64.02–2.60 (2.69–2.60)
Space group	*C*222_1_
*a*, *b*, *c* (Å)	77.9, 112.4, 140.7
α, β, γ (°)	90, 90, 90
Total No. of reflections	1562438 (150892)
Unique reflections	19282 (1865)
Overall multiplicity	81.0 (66.1)
Completeness (%)	99.24 (98.10)
Mean *I*/σ(*I*)	41.49 (1.65)
*R*_merge_	0.103 (2.760)
*R*_meas_	0.104 (2.781)
CC_1/2_	1 (0.75)
*R*_work_/*R*_free_	0.212/0.241
R.m.s.d., bond lengths (Å)	0.010
R.m.s.d., angles (°)	1.24
Ramachandran statistics	
Favoured (%)	98.5
Allowed (%)	1.5
Outliers (%)	0
Average *B* factors (Å^2^)	
Protein	
Chain *A*	87.3
Chain *B*	112.4
Chain *C*	167.9
DNA	
Chain *D*	94.3
Chain *E*	94.9
